# Do Unpaid Internships Breach Equality Law?

**DOI:** 10.1093/ojls/gqag018

**Published:** 2026-05-15

**Authors:** Joanna Helme

**Keywords:** internship, unpaid, equality, traineeship, discrimination, indirect

## Abstract

One of the pervasive legal questions surrounding unpaid internships is whether they are compliant with equality law. This speculation, however, has not yet faced judicial determination. This article analyses the merits of such a claim, focusing specifically on whether the EU recommendation that permits unpaid internships can be regarded as ‘invalid’ for discriminating based on social origin, age and sex. Whilst the arc of the article suggests that a successful claim is plausible, the analysis goes further by providing new light on quintessential debates within equality law. This includes the scope of the largely untouched issue of class discrimination; whether treatment can be both unfavourable and favourable simultaneously; the use of ‘common-sense’ deductions over statistics in indirect discrimination; and the concept of ‘broad discretion’ within the proportionality test. With millions of young people completing unpaid internships, tackling the question of their compatibility with equality law is of paramount importance.

## 1. Introduction

Within both public discourse and academic literature, there is now a substantial body of criticism regarding the discriminatory impacts of unpaid internships.^1^ However, the compatibility of such internships with equality law has not yet been comprehensively examined. With the vast majority of young people completing *at least one* internship in the EU, the force behind this question continues to mount.^2^ The purpose of this article is thus to examine what the role of equality law is, in light of the persistent criticisms, and determine to what extent equality law can address these concerns.

By way of background, internships are typically understood as a short period of work experience combined with a learning component, aimed at young people.^3^ The term ‘internship’, however, is poorly defined and, as a result, encompasses a diversity of arrangements. There are generally considered to be four types of internships: professional internships (those that are mandatory for a regulated profession, such as medicine or law); active labour market policy internships (for instance, where work placements are undertaken in return for unemployment benefits); student internships (those which are part of formal education and training curricula, typically a university placement); and open-market internships (which largely covers the stereotypical intern, working in an organisation to enhance their CV or secure a future job).^4^

There is debate over whether these categories are mutually exclusive or indeed exhaustive,^5^ but, of all the distinctions, the most controversial is the division between paid and unpaid internships.

Around half (45%) of all EU internships are unpaid,^6^ and in the UK specifically, 61% of interns receive less than minimum wage or no wage at all.^7^ At the same time, internships are rapidly becoming a prerequisite for many professions. Notable examples include the arts, law, construction, IT, finance, real estate and media,^8^ with 87% of new entrants to journalism completing an internship.^9^ The United Nations, White House, EU and UK Parliament all offer internships, making them a gateway to politics, and universities are increasingly reliant on internship ‘brokers’ to provide students with access to internships during their studies.^10^

The growth in internships reflects the importance of ensuring young people gain access to practical skills outside of traditional classroom-based learning.^11^ With youth unemployment rates at 15% in the EU,^12^ the use of internships to ‘bridge’ the gap between education and work has become an increasingly powerful tool within the labour market.^13^

However, the rise in these highly sought-after opportunities has, as mentioned, been met with concern that they are only accessible to some. The European Ombudsman, for example, labelled unpaid internships as ‘*de facto* social discrimination’ for excluding young people from poorer backgrounds^14^ and the UK’s influential Taylor Review stated that unpaid internships were ‘extremely damaging to social mobility’ and should be ‘stamped out’.^15^ There is substantial evidence to suggest that the bifurcation of paid and unpaid internships deepens racial inequality,^16^ with others labelling unpaid internships as ‘systematic age discrimination’.^17^ Given that unpaid interns are disproportionately women,^18^ with reports that over half experience sexual harassment,^19^ sex discrimination is also of concern, as is the risk of perpetuating disability discrimination, since reasonable accommodation rights only apply to workers.^20^

Despite this, there has not yet been a judicial determination over whether unpaid internships comply with equality law,^21^ and policy makers have not clarified the situation either. In the UK, for instance, Members of Parliament advertise unpaid internships^22^ despite consecutive government attempts to ‘crackdown’ on unpaid internships.^23^ The European Parliament has repeatedly ‘condemned the practice of unpaid traineeships as a form of exploitation’,^24^ yet the EU recommendation on internships allows interns to be unpaid.^25^

The issue is compounded by the fact that there is scarce legal academic literature on the lawfulness of internships. There are two main labour law sources on the regulation of internships within the EU,^26^ and only a handful of publications providing an overview of internship regulation from an international perspective.^27^ There has been no comprehensive legal analysis of the UK’s internship regulation^28^ and limited data collection on the issue by the UK government, EU or International Labour Organisation.^29^ Whilst the discriminatory implications of unpaid internships are well known, the avenues for challenging them have not been interrogated.

In order to address this lacuna, this article constructs a test legal case using EU equality law. Specifically, it examines whether Principle 8 of the EU’s Recommendation on a Quality Framework for Traineeships (QFT),^30^ which allows for internships to be paid or unpaid, can be regarded as ‘invalid’ for breaching the principle of non-discrimination (found in article 21 of the Charter of Fundamental Rights (the Charter)),^31^ in analogy with *Test-Achats*.^32^ The purpose of the article is not only to provide a practical answer to this question, but to interrogate several core debates within equality law. This includes examining the scope of *who* is protected by equality law; the constitutional peculiarities of the EU itself; the nature of ‘unfavourable’ treatment; the alternatives to statistical data in identifying indirect discrimination; and the robustness of counterfactuals as justifications for discrimination (ie that young people would be unemployed if not for unpaid internships).

Before beginning, however, three short explanatory remarks on scope must be made. The first is that the EU recommendation examined applies only to open-market and active labour market policy (ALMP) internships,^33^ so the article focuses exclusively on those two types. That said, however, these two types of internships are almost diametrically opposed. Many of the exclusionary impacts of unpaid open-market internships are not apparent in ALMP internships (since they are not strictly ‘unpaid’, nor aimed at young people).^34^ The article is thus predominantly focused on open-market internships. The caveat to this is that there are many instances where student internships, and occasionally professional ones, overlap with open-market internships.^35^ Where this is the case, it will be highlighted.

Secondly, the scope of the article is focused on *EU* equality law, and there are three predominant reasons for this. First, EU equality law forms the bedrock of many Member States’ approach to equality law. It is therefore likely that other jurisdictions would adopt a similar approach to the test case of this article. Second, the EU is currently drafting a new directive and recommendation on internships.^36^ Legal research into the compatibility of unpaid internships with non-discrimination law, given the existing dearth of literature or litigation, is therefore of potential utility. Lastly, a Court of Justice (CJEU) ruling on the compatibility of unpaid internships with EU equality law would have major ramifications for the millions of unpaid interns across Europe, since it would likely directly affect national legislation on internships. It is therefore the most impactful, strategic test claim to analyse.

As a final point on scope, the question of whether unpaid internships breach equality law is a *separate* question to whether unpaid internships are a lawful form of work. The issue has been addressed elsewhere^37^ and, whilst not the focus of this article, shows that equality law is just one avenue for analysing unpaid internships.

With this in mind, the structure of the article is as follows. It begins by framing the test legal case, determining the constitutional issues in bringing a claim before the CJEU. The next three sections then examine the merits of the case under the grounds of social origin, age and sex. The overall arc of the article is that unpaid internships could convincingly breach equality law on all grounds, but that bringing a claim would spark multiple debates within equality law. The need for robust, clear legislation on unpaid internships is therefore paramount.

## 2. Framing the Claim: Constitutional Issues

Given the sensitivity around internships,^38^ the EU currently regulates the issue using ‘soft’ law. As this section will demonstrate, bringing a constitutional challenge against such regulation is difficult—although not impossible.

As it stands, there is very limited regulation of internships at an EU level. The only EU instrument which directly addresses internships is the non-binding Recommendation on a Quality Framework for Traineeships, issued in 2014,^39^ which is currently under review.^40^

The QFT, as mentioned, does not cover student or professional internships. Instead, it provides guidance to Member States on open-market and ALMP internships by outlining 21 good-practice principles. These include the use of a written agreement for internships, transparent advertisements and limiting internships to a reasonable duration. The QFT, however, is more notable for what it *does not* recommend than for what it does. The Recommendation does not clarify the employment status of interns, nor recommend that they be paid. Principle 8 QFT instead states that the internship agreement should ‘clarif[y] whether an allowance or compensation is applicable, and if applicable, its amount’. The provision therefore had the effect of legitimising the use of unpaid open-market internships across the EU,^41^ whilst also creating two types of internships: paid and unpaid.

The most impactful claim that could be brought before the CJEU would be to challenge the QFT directly, by arguing that the provision of unpaid internships in Principle 8 QFT breaches the principle of non-discrimination in article 21 Charter, which states that:

any discrimination based on any ground such as sex, race, colour, ethnic or social origin, genetic features, language, religion or belief, political or any other opinion, membership of a national minority, property, birth, disability, age or sexual orientation shall be prohibited.

Several grounds could be put forward, but the most relevant are social origin, age and sex (for reasons discussed subsequently).

Under EU law, there are two possible avenues for reviewing the compatibility of an EU provision with the Charter: either through an action for annulment under article 263 TFEU^42^ or through a preliminary ruling under article 267 TFEU.

Whilst an action for annulment would be the most traditional route, there are two main obstacles under article 263 TFEU: the time limit and the explicit exclusion of reviewing ‘recommendations and opinions’.

Neither of these hurdles is insurmountable, however. The QFT is currently being updated by the Commission, thus reopening the time limit if adopted, and the CJEU has made inroads into the restriction on reviewing recommendations by applying it only to ‘genuine’ recommendations.^43^ There is scope to argue that the QFT is *not* a genuine recommendation if it is considered to contain mandatory language and an intention to have legal effects.^44^ This may be particularly true for the updated QFT, which comes as a ‘package’ with the directive,^45^ suggesting that they work in tandem, especially given that national courts are already required to consider recommendations in their decision making.^46^

What is more significant is whether there *ought* to be an exception to the bar on the reviewability of recommendations if they *prima facie* breach a fundamental principle of EU law such as non-discrimination. There does not appear to be a CJEU ruling on this issue; however, it seems illogical that recommendations could provide a *carte blanche* for the EU to act contrary to the Charter or general principles of EU law without CJEU oversight.^47^

The issue is of heightened concern because the Commission’s new proposals continue to rely on a recommendation to regulate internships (the proposed directive only applies to interns with worker status). One of the most cited reasons for this is that the EU lacks the competency to issue directives on education and vocational training.^48^ Whilst this competency debate is beyond the scope of this article, it has been argued that the ‘centre of gravity’ (the CJEU’s test for determining which treaty basis should be used)^49^ is *working conditions* under article 153(1)(b) TFEU rather than *education*. This is because there is no robust regulation of the educational aspect under the QFT (nor the updated proposals), thus the aim of education is not guaranteed.^50^ The overarching point, however, is that where recommendations are used to potentially diffuse the controversy around the fundamental rights at stake (such as the right to pay or non-discrimination), then they ought as a matter of principle to be reviewable.

Given the hurdles faced under article 263 TFEU (ie the exclusion of recommendations and the time limit), it seems more viable to review the QFT through a preliminary ruling under article 267 TFEU. This allows national courts to refer a question to the CJEU on the validity and interpretation of ‘acts’ of the EU, which includes recommendations.^51^ This then represents a further constitutional peculiarity, which is the potential backdoor annulment of a recommendation through a preliminary ruling. The CJEU has, however, allowed for preliminary rulings to be delivered even where an overlap with annulment actions exists.^52^

As such, a question on the compatibility of the QFT with article 21 Charter could find its way before the CJEU as a preliminary ruling. There are different avenues in which this could occur, the most foreseeable being a dispute over any national law that implements the QFT. This scenario would be analogous to cases like *Test-Achats*, *Digital Rights Ireland*,^53^  *Volker*^54^ and *Schrems*,^55^ where the referring court asked whether EU law (or the national law that transposed EU law) was compatible with certain Charter provisions. This formulation avoids having to examine whether the case falls within the scope of the Charter or if article 21 has direct effect, as the question turns on the compatibility of EU law with the Charter. Applied to unpaid internships, the question would be whether the national law that implements the QFT (or the QFT itself) infringes article 21 Charter. This framing would nevertheless have the curious consequence of a Member State being found in breach of the Charter for implementing an EU recommendation.

This section has thus highlighted the possible avenues for examining the QFT’s validity, with a preliminary ruling being the most foreseeable. It has also flagged future constitutional issues to consider as the Commission updates the QFT, based on the legal effects of recommendations and the liability of Member States for acts of the EU.

With the framing of a claim laid out, this article now turns to examining the substantive merits based on three grounds. The essential question posed is, do unpaid internships discriminate against the working class, young people and/or women—and thus, should Principle 8 QFT be held as invalid under article 21 Charter? Each ground will be examined in turn, starting with social origin.

## 3. Do Unpaid Internships Discriminate Based on Social Origin?

Social origin, often discussed in connection with ‘class’, ‘socio-economic’ or ‘poverty’ discrimination,^56^ is an underdeveloped and controversial area of equality law.^57^ There are almost no supranational human rights decisions on social origin^58^ and few Member States recognise it as a ground of discrimination.^59^

Despite this, social origin discrimination is central to the debate around unpaid internships. The QFT itself concedes that unpaid internships ‘may limit the career opportunities of those from disadvantaged backgrounds’^60^ and the European Parliament has argued that they ‘unfairly exclude’ those from vulnerable backgrounds.^61^

A claim before the CJEU that Principle 8 QFT breaches article 21 Charter would therefore provide a unique, landmark opportunity to develop jurisprudence on social origin discrimination. This is because the Charter explicitly prohibits discrimination based on social origin, and the viability of unpaid internships is closely tied to social origin.

The starting point is to ask whether the CJEU would contemplate engaging with social origin under article 21 Charter, given how unprecedented it would be. One issue concerns defining social origin,^62^ which the CJEU has not done yet.^63^ It seems plausible, though, that the Council of Europe’s definition would be adopted;^64^ it considers social origins as the ‘social or class milieu into which a person is born and that shapes their formative years: their origins, upbringing or starting point in life’.^65^ This is distinct from ‘socio-economic status’, which refers to a person’s *current* situation (although the two often coincide).^66^ In the context of unpaid internships, the CJEU might further consider social origin in conjunction with the interrelated ground of ‘property’,^67^ given the focus of discrimination based on financially disadvantaged social origins (ie a lack of financial property).^68^

There are several reasons to think that the CJEU might engage with social origin discrimination. The first is that unpaid internships appear to provide an ideal, pioneering test case for social origin discrimination. The anticipated pitfalls of a social origin claim are absent: young interns are still proximate to their social origins, having not had the opportunity for social mobility, and the requirement for accessing unpaid internships rests firmly on financial resources, as opposed to more tangential class indicators (like accent, education or region). Floodgate concerns can also be mitigated due to the relatively self-contained nature of the claim. This is because of the distinct role internships play as a ‘bridge’ between education and work, which is critical for social mobility. Comparison is thus hard to draw with other social origin discrimination claims based on the unaffordability of goods or services, due to the design of internships as a portal to employment (this point will be returned to later).

The second reason why the CJEU might be inclined to engage with social origin is timing. As Malleson has argued, socio-economic discrimination is one of the fastest growing axes of discrimination today. This rise in wealth inequality has been paralleled with a push-back against anti-discrimination efforts and right-wing politics in Europe.^69^ Bringing the so-far-dormant ground of social origin discrimination into the frame may be highly informative in shaping the future of equality law. This is particularly compelling in an EU context, where there is an explicit focus on combatting not just discrimination but ‘social exclusion’,^70^ which has been interpreted as related to social mobility.^71^

Thus, given that the CJEU has regarded the Charter as a ‘living instrument which must be interpreted in light of present-day conditions and of the ideas prevailing … today’, there may well be reasons to believe it would engage with social origin as a ground.^72^ Assuming that the CJEU *does* acknowledge social origin, the next stage of analysis is whether discrimination can be made out.

Like most equality law frameworks, the EU distinguishes between direct and indirect discrimination. Direct discrimination occurs when a person is treated less favourably than a comparable other based on protected grounds, whilst indirect discrimination occurs when an apparently neutral provision puts persons of a protected ground at a particular disadvantage in a way that cannot be justified objectively.^73^ The distinction is critical because only indirect discrimination can be justified: ensuring the boundary is fairly drawn between the two is a quintessential debate within equality law.^74^

It seems fairly apparent that a *prima facie* indirect discrimination claim based on social origin can be made out. Focusing on Principle 8 QFT’s provision of unpaid open-market internships, whilst this rule appears neutral, studies consistently show that unpaid internships put those from less financially advantaged backgrounds at a particular disadvantage compared to those from wealthier backgrounds.^75^ In order to complete the unpaid open-market internship, the intern will need financial support from alternative sources. Families or friends that can provide financial support for living costs (including the internship brokers’ fee, if relevant) and/or accommodation where the internship is located would significantly aid the intern’s ability to complete one. The importance of social origins is furthered by the fact that many internships are not advertised but offered through social connections, with recent UK data showing that only one in ten internships were openly advertised.^76^ A lack of both financial support and social connections arising from the intern’s social origins may thus dramatically affect their ability to access unpaid internships.^77^

An initial finding of *indirect* discrimination therefore seems straightforward: this has been confirmed by the European Ombudsman’s decision which claimed it was ‘undeniably’ easier for young people from wealthier backgrounds to complete unpaid internships.^78^

What is more uncertain is whether a *direct* discrimination claim could be made out. Although Principle 8 QFT does not explicitly ban people from certain social origins from taking part, a line of CJEU jurisprudence leaves scope to argue that it could still be directly discriminatory.

The CJEU has held that criteria which are ‘inseparably’ or ‘inextricably linked’ to a protected characteristic;^79^ affect ‘in whole or in part’ a protected group;^80^ or are ‘based primarily’ on a protected characteristic^81^ can still amount to less favourable treatment required for direct discrimination. Unpaid internships can be analogised to these ‘proxy’ discrimination cases, where a seemingly different criterion is used that is almost directly linked to the protected characteristic.^82^ This is because the very criterion for undertaking an ‘unpaid’ internship rests directly on the intern’s financial resources. Given that social origin concerns ‘those who start out in life less well off’, as defined by the Council of Europe,^83^ this makes the provision of unpaid internships ‘inextricably linked’ to the protected characteristic of social origin combined with financial property. While some interns from poorer social origins may be able to afford unpaid internships (eg by working weekends), participation is ‘primarily based’ on the resources of one’s social origin, and the pool of those who can participate is ‘in whole or in part’ made of young people from wealthier social origins.^84^ There is therefore scope to argue that the explicit division between paid and unpaid internships acts as a proxy criterion which directly discriminates along social origin lines. This would then speak more broadly to the distinction between direct and indirect effect: although direct discrimination aims to reflect the disparate *treatment* of others while indirect discrimination is *effects-based*,^85^ if the *foreseeability* of unpaid internships discriminating based on social origin is clear, then the division between the two types is less apparent.

There are thus different considerations for whether the CJEU would find Principle 8 QFT directly or indirectly discriminatory. Under both avenues, however, it seems plausible that the CJEU would nevertheless consider justifications under article 52 Charter, as it did in *Test-Achats*.^86^

The question of whether unpaid internships can be justified goes to the heart of the case on all grounds, not just social origin. It is the most controversial aspect of the claim, as it essentially asks what level of discrimination society can tolerate if it achieves an alternative aim. The legal test is whether the discrimination is justified by a legitimate aim achieved through suitable and necessary means.^87^

Step one is therefore to establish the legitimate aims being pursued by the provision of unpaid internships in Principle 8 QFT. The most frequently cited aim of internships—regardless of type or remuneration—is to generate *practical* experience (in contrast to classroom-based learning), which leads to the interrelated aim of improving young people’s entry into the labour market.^88^ This seems likely to qualify as a ‘legitimate’ aim since it has been accepted previously,^89^ and the CJEU tends to take a lenient approach.^90^

Step two is to determine whether Principle 8 QFT is a suitable means of achieving this aim. This involves an interrogation of whether unpaid internships actually lead to practical skills acquisition and, ultimately, employment.

Several arguments can be put forward to support this. By not requiring payment, Principle 8 QFT roots the ideology of internships firmly in education and the bestowing of practical experience, rather than employment. If payment were part of the internship criteria, then the internship providers’ focus may be distorted towards productivity and deliverables as opposed to training for training’s sake.

By not providing payment, Principle 8 QFT also helps break the cycle of graduate students needing experience in order to get a job, yet not being able to find experience without a job.^91^ The alternative might be volunteering, which is far less likely to be structured, openly advertised or regulated when compared to internships that comply with the QFT. Particularly for university students, the use of internship intermediaries to provide open-market summer internships might reduce the time spent sourcing internships, thus lessening the stress of balancing education with securing experience. Lastly, for professions where practical skills acquisition is *not* a mandatory requirement, but clearly beneficial educational courses are—examples might include hairdressing, mechanical engineering, geology and events management—the existence of a quasi-regulated unpaid internship may be a suitable means of fortifying classroom learning *without* making it a regulated profession.

The challenges to the suitability test, however, rest largely on the lack of evidence that unpaid internships do lead to the legitimate aims outlined. In terms of skills acquisition, studies consistently show that *paid* internships are the most effective means of ensuring quality training, possibly because internship providers have a vested interest in ensuring training actually occurs.^92^ For ALMP internships, it has been argued that skills acquisition is rarely the central purpose; instead, the internship is perceived as a mechanism for the intern to ‘earn’ their unemployment benefits and ‘give back to society’.^93^ There is also a concern that open-market internships are geared towards providing a foothold in a *specific* firm, hence the skills learnt are not necessarily transferable.^94^

Evidence also indicates that unpaid internships are not necessarily a suitable means of stimulating youth employment.^95^ This is built on the premise that if insufficient jobs are available, then the provision of unpaid internships does not fundamentally alter that fact. Instead, unpaid internships may be achieving the opposite effect by becoming an entrenched prerequisite to employment, which prolongs the job-hunting period.^96^ Support for this is indicated by the Eurobarometer finding that over half of the 26,000 surveyed completed *multiple* internships: it seems perhaps unconvincing to claim that unpaid internships facilitate entry into the labour market if multiple are required.^97^ The CJEU has often stressed that ‘mere generalisations’ that a measure contributes to the labour market or vocational training are ‘not enough’ to justify discrimination and ‘do not constitute evidence’,^98^ and it seems plausible that the need for robust evidence is particularly crucial for unpaid internships, given their implications on social mobility.

If, nonetheless, Principle 8 QFT were regarded as a suitable measure, then attention would turn to the second limb of proportionality—the necessity test—which seems likely to be the most contentious part. It would need to be shown that Principle 8 QFT is necessary for securing practical experience and youth employment, despite the discriminatory impact on social origin.

The central argument here is that, were Principle 8 QFT to require payment, then many internships would no longer exist. The costs and obligations involved in providing quality traineeships—namely mentorship, setting learning objectives and dedicating staff time to support the intern—on top of providing remuneration may render internships an unattractive, cumbersome option. In such circumstances, it would appear preferable to hire a fixed-term worker without the additional learning and training requirements over a paid intern. The argument is particularly stark for ALMP internships, where the intern, unlike a graduate intern who is seeking a specific job, has struggled to get *any* job and therefore the internship provider may be less incentivised to take them on if remuneration is required.^99^ Comparison also cannot be drawn with the introduction of minimum wage legislation (where it was incorrectly claimed that doing so would reduce the number of jobs^100^) because quality unpaid internships should not be *replacing* entry-level jobs. If, for example, a bike mechanic needed an extra pair of hands, then this should be a fixed-term job, not an internship. If, however, the bike mechanic was keen to show to the community that she supports local young people or simply wanted to pass on her trade, then this would be an internship. Clearly, the latter would likely disappear if payment was required whereas the former would not. Hence, it can be argued that Principle 8 QFT is necessary, despite the discriminatory implications, to ensure genuine internships exist.

By contrast, it can be argued that Principle 8 QFT is *not* necessary to achieve the set aims on two fronts. The first is to argue that less restrictive means could have been used and that the blanket allowance of unpaid internships under Principle 8 QFT appears disproportionate. In the age discrimination cases of *Mangold* and *Petersen*,^101^ the CJEU held that the failure to consider personal situations or the specificities of the labour market rendered a measure disproportionate. Principle 8 QFT, however, contains no criteria around the use of unpaid internships. There is no distinction based on the interns’ personal circumstances, the specificities of the labour market or the interns’ entitlement to alternative funds. For instance, unpaid internships can be used in regional labour markets where there is high youth *employment*: in these circumstances, Principle 8 QFT is not facilitating entry but potentially stifling access to regular employment. Similarly, Principle 8 QFT could have qualified the use of unpaid internships to circumstances where alternative funds are available (such as government or university scholarships) or, where no funding is available, limited the duration to four weeks to lessen the barrier to access.^102^ The lack of qualifiers around Principle 8 QFT which could mitigate the impact on social origin discrimination suggests a failure to balance the competing aims. This is furthered by the lack of evidence demonstrating that requiring open-market internships to be paid would reduce the number offered: in the UK’s leading empirical study on internships, for instance, three-quarters of employers stated that a ban on unpaid internships would not impact the number they provided.^103^ Since many internship providers use internships as an effective screening mechanism for future employees, it is not necessarily compelling to claim that open-market internships would disappear if payment was required.^104^ In any event, it is certainly tenuous to argue that a lack of payment is *necessary* to facilitate youth employment.

The second line of counterargument is to claim that the need to prevent the discriminatory impacts of unpaid internships outweighs the potential (largely unevidenced) gains made to youth skills and unemployment.

The starting point is to recognise the importance of a job, unlike other opportunities which may be inaccessible due to costs (such as products, holidays or even housing),^105^ in addressing social exclusion and ensuring social mobility.^106^ Education, as the ‘great equalizer’, is presented as the key mechanism for accessing jobs which facilitate mobility outside of one’s social origins.^107^ Whilst there is much debate over whether education does achieve this,^108^ the principle of accessible education is critical to justifying society’s social inequality.^109^ This is why unpaid internships are particularly controversial because their characterisation as a ‘bridge’ between education and employment undermines both as vehicles for social mobility. If this bridge is not accessible, then there is a real risk that unpaid internships will lock out socially disadvantaged young people from achieving social mobility regardless of their meritocratic ability. In the case of the bike mechanic, if an intern cannot complete an internship due to the expense, yet completing an internship is a ‘must’ for becoming a bike mechanic, then the benevolence of offering genuine internships to support the local community appears to lose its good intentions.

This is perhaps best illustrated through discussion of professional internships (which are excluded from the QFT). In this type of internship, the need for practical experience is deemed so important that it is mandated by national regulation. Hence, for professions such as surgery, dentistry, nursing, electricians and firefighters, it could most starkly be argued that the integrity of the role requires practical experience, thus outweighing the discriminatory implications of unpaid internships on social origins. And yet, professional internships are the most likely to be paid.^110^ Gatekeeping public service sectors like medicine, teaching and firefighting from those with financially disadvantaged social origins may be regarded as untenable for social cohesion. It undermines the national educational system if, regardless of academic achievement, extended periods of unpaid work are mandatory for foundational careers. Hence, by comparison to professional internships, where the need for practical experience is most compelling, it seems *even harder* to justify unpaid open-market internships, where practical training is not deemed by national regulators as necessary to the trade. This is especially the case where unpaid open-market internships are being used as a vetting process for accessing paid professional internships (such as legal internships prior to a training contract^111^), since it undermines the regulatory requirement for those qualifications to be remunerated.

In light of these points, the CJEU may consider the indiscriminate use of unpaid open-market internships in Principle 8 QFT disproportionate relative to the aims pursued. Such a finding would not be without support: the Council of Europe has issued a resolution calling for Member States to consider banning unpaid internships due to social origin discrimination^112^ and the European Ombudsman has declared their use as ‘maladministration’ for perpetuating social exclusion.^113^ It is thus certainly feasible that Principle 8 QFT could be held as ‘invalid’ for discriminating based on social origin under article 21 Charter.

If, however, the CJEU does not find Principle 8 QFT to discriminate on the ground of social origin, then it is still possible that other grounds could be made out.

## 4. Do Unpaid Internships Discriminate Based on Age?

Unpaid internships are aimed at young people, making them susceptible to an age discrimination claim. It could be argued that the lack of remuneration for interns under Principle 8 QFT amounts to less favourable treatment based on age under article 21 Charter.

There are three main CJEU cases, however, which could disrupt a finding of age discrimination: *O*,^114^  *Abercrombie*^115^ and *Hubertus*.^116^ This section will examine each case in turn, interrogating three thematic issues: whether treatment can be both ‘favourable’ and ‘unfavourable’ simultaneously; whether there is an adequate comparator for young people; and whether there should be ‘broad discretion’ to justify age discrimination.

Before analysing the three cases, however, a preliminary remark should be made on whether the CJEU would analyse internships under direct or indirect age discrimination. Age is characterised as a unique ground in non-discrimination law because everyone should, at some stage, be ‘old’ or ‘young’.^117^ There are thus milestones associated with each age group that might warrant different treatment. Whilst this characterisation is highly disputed,^118^ it is nevertheless the framework adopted by EU equality law. As a result, *direct* age discrimination can be justified, unlike other grounds.^119^

This means that the distinction between direct and indirect age discrimination is potentially less significant. However, for the purposes of internships, the distinction is critical. This is because internships are increasingly promoted as *not* age specific, but as a broader lifelong learning opportunity for all ages.^120^ The issue is compounded by the fact that student internships are the most common type of internship in the EU,^121^ meaning that mature university students will be affected by any age restrictions.^122^

Under the QFT, the definition of internships does not explicitly reference age, so it could be argued that they are not confined to young people. However, in practice, internships are designed for young people. The QFT preamble refers multiple times to the plight of young workers; the EU data on internships addresses 18-to-35-year-olds;^123^ and some definitions in EU legislation refer to those exclusively under 30 years.^124^ Internships might therefore be better regarded as a proxy for age, and thus directly discriminatory. Given the CJEU’s reliance on proxies within age discrimination,^125^ this seems highly plausible.

The reason this distinction is important is because, if internships are promoted as *not* age specific, then the justifications for their existence (eg alleviating youth unemployment) are no longer tenable. This was the case in *Hütter*, where the CJEU held that a measure which did not consider the age of the apprentice could not then be justified on the basis of promoting young people’s entry into the labour market (a similar point was also made in *Kücükdeveci*^126^).^127^ With this in mind, the analysis now turns to examining the three contested CJEU cases.

### A. Unfavourable Treatment and *Hubertus*

For there to be discrimination, a person of a protected characteristic must be ‘treated less favourably’ or put at a ‘particular disadvantage’ compared to others.^128^ The difficulty with unpaid internships, however, is that there is mixed data on whether they are in fact beneficial to the young person. Some studies have highlighted *negative* impacts on job opportunities, especially when compared to paid internships.^129^

This issue becomes problematic when examining different grounds of discrimination. A discrimination claim based on social origin assumes unpaid internships are advantageous, whereas a claim based on sex makes the opposite assumption—it argues that the overrepresentation of women in unpaid internships compared to men is detrimental to their career.

The disagreement over whether unpaid internships are unfavourable or not becomes most apparent when considering age discrimination. This is because age discrimination claims against internships work both ways. There are cases in the United States of *older* workers bringing discrimination claims for being denied internships due to their age,^130^ based on the assumption that unpaid internships are advantageous.

Analogy could be made with the decision in *Hubertus*, where the CJEU held that there was no age discrimination where contracts could be extended for retired workers, if they desired, because it was not ‘unfavourable’ treatment.^131^ Similar remarks were made in *Georgiev*, concerning comparable facts.^132^ Building on this, it could be argued that unpaid internships are not unfavourable because they benefit young people by providing them with work experience. In this sense, they may look more like positive action: unpaid internships are designed to *advance* young people onto the labour market. It could further be argued that it would run amok of equality law to find that unpaid internships are both favourable and unfavourable at once.

The rebuttal to this argument (that unpaid internships are favourable) is twofold. The first point to note is that the disadvantageous impact of unpaid internships can be felt by multiple age groups contemporaneously: older workers may struggle to access certain professions without internships, while young people may feel pressured to work without pay to access these professions. Multiple claims attest to the multifaceted, unregulated issues with unpaid internships, rather than undermining non-discrimination law.

The second point of rebuttal relies upon the Advocate General Opinion in *Abercrombie*.^133^ This case concerned an Italian law which authorised the use of on-call contracts for under 25-year-olds in all circumstances (whereas other age groups could only conclude on-call contracts in limited circumstances). The issue of less favourable treatment had ‘given rise to quite some discussion before the Court’, according to the Advocate General.^134^ Abercrombie argued that the law resulted in *more favourable* treatment of young workers by granting them a ‘privileged contractual status’ which made them ‘more attractive for employers’.^135^ Whilst the CJEU did not address the issue, the Advocate General dismissed this reasoning. He argued that ‘hardly any measures could be said to be exclusively favourable’ and that there should be a ‘comprehensive assessment’ of the measure, with the point of reference being the ordinary employment relationship.^136^ Following this, it could be argued that unpaid internships, compared to standard employment, are unfavourable. The lack of payment, employment rights and security associated with unpaid internships are all issues which the CJEU has previously considered disadvantageous.^137^ Moreover, it arguably goes against the grain of employment law to argue that a lack of worker rights is somehow a ‘privilege’: this risks lowering, and possibly destabilising, the floor of agreed protections.

### B. Comparator and *O*

The next major hurdle to an age discrimination claim is finding a relevant comparator. The role of a comparator in EU equality law has long been controversial as a potential hinderance to substantive equality,^138^ and unpaid internships attest to this issue.

In *O*, the CJEU held that the exclusion of ‘young people’ working during their school or university vacation on fixed-term contracts from the end-of-contract payment granted to other fixed-term workers was not discrimination.^139^ The student workers were not in an objectively comparable situation to other workers, and therefore the difference in treatment was not age discrimination. A similar line of argument was seen in *Wippel*, where the CJEU held that a sex discrimination claim could not be made between a regular worker and an on-demand worker, as the contracts were too different to compare.^140^

From this, it could be argued that internships are unique contracts, designed for learning and training rather than remuneration, which assist young workers in the education-to-work transition. The situation of young people, facing structural unemployment which internships attempt to alleviate, constitutes a ‘fundamental distinguishing element’ from other age groups.^141^ There are therefore material differences, outside of age, that make comparison with older, regular workers untenable.

Several arguments can be made to rebuke this assertion. The first is that, following this logic, the CJEU ought not to be able to compare older workers with other age groups due to their unique set of circumstances. The CJEU has acknowledged that older workers ‘encounter considerable difficulties in finding work’^142^ and, ‘unlike young workers’, workers of retirement age have the choice between continuing to work or retiring.^143^ Moreover, contracts specifically designed for older workers facing retirement, seen in the CJEU cases of *Mangold*, *Georgiev* and *Hubertus*, could be regarded as ‘work-to-retirement’ transition contracts, which are not comparable to other contracts, in analogy with ‘education-to-work’ transition internships.

However, since the comparator test has *not* been an issue in these older worker cases, it would be inconsistent to approach young workers cases differently.

A further line of rebuttal would be to draw a comparison based on workplace training. If the issue is that internships are not comparable to fixed-term contracts because the role itself is different, then comparison could instead be made between regular workers undertaking workplace training and interns, given that internships are couched as a learning and training opportunity.^144^ The question then becomes, why are unpaid internships treated differently compared to other age groups receiving workplace training? *De Lange* could be relied upon to support this, in which the CJEU held that a Dutch taxation measure which deducted the full costs of vocational training for workers under 30, but not those over 30, was not discriminatory.^145^ The CJEU endorsed the Dutch government’s argument that young people were *less* able to bear the financial burden of training than older workers because they had recently left the school system. It would therefore be contradictory for the CJEU to hold the opposite: that young people were better able to afford the unpaid training during an internship than their older counterparts.

The third and final rebuttal is that the CJEU in *O* arguably turned the comparator test into a justification test. The CJEU claimed that the young students were not comparable ‘in terms of insecurity’ to other fixed-term workers since their jobs were ‘temporary and ancillary’.^146^ However, these factors arguably justify why the students were excluded from the end-of-contract payment: they do not negate the fact that the young workers were treated differently. More fundamentally, it seems counterintuitive that a policy which exclusively targets one group is shielded from non-discrimination law *precisely because* the disadvantaged is so confined to a specific group that it cannot be compared. This line of reasoning suggests that the more entrenched and structural the discrimination is, the less likely the group will be afforded equality law protection as comparison becomes too fraught. It is therefore preferable, as a matter of principle, that these issues are considered in the justification stage, not the comparator stage.

### C. Justification and *Abercrombie*

Direct age discrimination can be justified, which marks the last major hurdle to a claim that Principle 8 QFT discriminates based on age.

Since many of the arguments considered under the social origin claim transfer across to this claim, they do not bear repeating here. However, there is one case which specifically relates to youth discrimination which should be considered.

As mentioned earlier, the CJEU in *Abercrombie* held that a law allowing for the unrestricted use of on-call contracts for under 25-year-olds was justified because it pursued the legitimate aim of facilitating young people’s entry onto the labour market. The CJEU held that law was appropriate and necessary in light of Italy’s low youth unemployment rate, accepting the arguments that the on-call contracts were ‘preferable’ to unemployment; that they acted as a ‘springboard’ into stable employment; and that employers would not be able to offer the contracts to the same number of young people otherwise.^147^

All of these arguments pertain to Principle 8 QFT, which also intends to facilitate labour market transitions. Considering Europe’s high youth unemployment, it can likewise be argued that unpaid internships are preferable to no experience and that requiring them to be paid would reduce the number offered. This can be fortified in light of the Equality Framework Directive, which explicitly refers to ‘remuneration conditions’ as an example of potential differences in treatment based on age,^148^ and the comments in *Hütter*, which tentatively suggest that the CJEU is not, in principle, opposed to a lower national minimum wage for young workers.^149^ It could therefore be argued that in the specific context of age discrimination, the provision of unpaid internships under Principle 8 QFT is justified.

However, there is reason to doubt whether the CJEU would adopt the approach seen in *Abercrombie*. In that case, the CJEU placed significant weight on the ‘broad discretion’ given to Member States to justify age discrimination (a sentiment reflected in other cases too).^150^ This discretion is indicative of the unique characterisation of age when compared to other grounds.^151^ As a result, the CJEU dealt only cursorily with the claims in *Abercrombie* without interrogating the evidential support behind them. However, in circumstances where the CJEU is evaluating an EU measure (in this case, Principle 8 QFT), the rationale for providing a broad discretion is lacking. This is because the EU is making a *recommendation*, which Member States retain general discretion to implement. It thus seems plausible that the CJEU would pay closer attention to the proportionality requirements of Principle 8 QFT than it did in *Abercrombie*, to ensure that the advisory recommendation made by the EU is warranted despite the potentially discriminatory effect.

Were the CJEU to engage in the proportionality test, it seems possible that Principle 8 QFT would struggle under scrutiny. This is because the CJEU, in general, has taken a ‘robust’ approach to age discrimination, despite its characterisation as a unique ground in equality law.^152^ As O’Cinneide notes, the CJEU has leaned towards treating age similarly to ‘suspect’ grounds, like sex and race.^153^ Given that it would be ‘unthinkable’ to lower wages to improve the labour market participation of women or ethnic minorities,^154^ there is reason to doubt whether the CJEU would readily accept Principle 8 QFT in the same manner as seen in *Abercrombie*. It seems probable that greater interrogation of the evidential support for the claim that unpaid internships act as a ‘springboard’ to employment or that payment would result in reduced opportunities would be needed, as required in other age discrimination cases.^155^ Given the indiscriminate use of unpaid internships under Principle 8 QFT, this may also be challenged in line with age discrimination cases that have criticised measures which result in unequal treatment *within* young people.^156^ For example, Principle 8 QFT does not distinguish within the young age bracket despite the likelihood that an unpaid intern in their early 30 s may feel the hardships of non-remuneration more acutely than an unpaid teenage intern still living with their parents. It also seems probable that a distinction between *Abercrombie* and a Principle 8 QFT claim can be made on the basis that the severity of the hardship associated with unpaid internships appears *greater* than the unrestricted use of paid on-call contracts for under 25-year-olds. Hence, in a scenario where the EU is making a *recommendation* to Member States, it seems likely that the CJEU would place greater emphasis on the proportionality test when examining Principle 8 QFT, as analysed in the social origins section.

To conclude this overview, there are ultimately various ways in which an age discrimination claim could be made out before the CJEU, albeit with several hurdles. This would largely depend upon the three main cases identified—*Hubertus*, *O* and *Abercrombie*—being distinguished.

Analysis now turns to the final ground of discrimination, to determine whether unpaid internships can be considered discriminatory based on sex.

## 5. Do Unpaid Internships Discriminate Based on Sex?

Women undertake a disproportionate amount of unpaid work compared to men,^157^ and it appears that internships are no different. This section examines Principle 8 QFT in light of this, focusing specifically on the CJEU’s alternative approaches to demonstrating indirect discrimination and the EU’s (overlooked) mainstreaming obligations.

According to recent EU data, 60% of male interns are paid compared to 49% of female interns.^158^ This trend is reflected in other studies,^159^ some of which have found that as high as 77% of unpaid interns are women.^160^ The apparent overrepresentation of women in unpaid internships has caused concern because not only does it potentially entrench the gender pay gap,^161^ it also risks generating a gender ‘rights’ gap. Since paid internships are more likely to come with worker status,^162^ women appear to be disproportionately entering the labour market with *neither* pay *nor* employment rights. This is then compounded by the fact that paid internships are associated with better labour market opportunities post-internship,^163^ potentially resulting in reduced access to future employment for women compared to men.^164^

Even when comparing between unpaid male and female interns, there is evidence to suggest that disadvantages of being unpaid are felt more harshly by women. Whilst women are globally 64% more likely to experience sexual harassment at work than men,^165^ studies suggest unpaid interns are at ‘disproportionate’ risk of sexual harassment because of their intersecting statuses.^166^ The combination of youth; inexperience; the age and power disparity with seniors; the precarity of having limited workers’ rights,^167^ trade union protection or knowledge of reporting mechanisms; the fear of losing a potential job opportunity; as well as a high grade (in the case of student interns) are all contributing factors that make unpaid female interns particularly vulnerable to abuse.^168^ Rates as high as 75% of female interns experiencing sexual harassment have been reported,^169^ and cases like *Wang* in the United States, in which a female intern alleging sexual assault by her internship supervisor had no recourse to employment law, underscore these risks.^170^ Given that unpaid interns are hoping, in the words of the UK Supreme Court, to ‘impress’ their prospective employer,^171^ they may also be less likely to complain of sexual harassment for fear of harming their career prospects.

Translating these concerns into a legal claim could take two forms. One argument would be that Principle 8 QFT indirectly discriminates based on sex under article 21 for allowing both unpaid and paid internships. Whilst this provision appears neutral, the bifurcation puts women at a particular disadvantage because they are more likely to be unpaid. Analogy can be made with CJEU cases concerning part-time and full-time workers.^172^ In these cases, the CJEU has considered the fact that, since women are significantly more likely to be part-time workers, the benefits afforded to full-time but not part-time workers can amount to indirect sex discrimination. It could therefore be argued that the benefits given to paid interns (namely remuneration and, typically, worker rights) compared to unpaid interns amounts to indirect sex discrimination.

The primary challenge with this claim is whether there is sufficient evidence to demonstrate that those suffering the disadvantage are predominantly women. How the CJEU determines whether there is a ‘particular disadvantage’ has long plagued EU equality law^173^ and, in the case of indirect sex discrimination specifically, the situation is further complicated by the intertwined discussion of equal pay and part-time work discrimination, which have separate provisions to sex discrimination.^174^

In general, however, the CJEU has two approaches for establishing a particular disadvantage: either applying a strict statistical approach or adopting a ‘common-sense’ approach.^175^ Historically, the CJEU has relied upon the statistical approach to demonstrate indirect sex discrimination;^176^ whilst legislative changes attempted to shift this towards lesser reliance on statistics,^177^ the use of statistics has persisted.^178^

The statistical approach, asserted notably in the cases of *Seymour-Smith*,^179^  *Jenkins*,^180^  *Bilka*,^181^  *Jørgensen*^182^ and *BVAEB*,^183^ requires evidence that a ‘considerably smaller proportion of women than men’ are in the advantageous group, or that a ‘persistent and relatively constant disparity’ between men and women exists.^184^ Case law on this approach indicates that around 80–90% of the disadvantaged group should be comprised of women to successfully demonstrate a particular disadvantage.^185^

If this strict statistical approach is adopted, a sex discrimination claim against Principle 8 QFT would be difficult to sustain. The existing EU data seems unlikely to meet the threshold under the CJEU’s case law, and even though some statistics are nearer the 80% mark, the lack of long-term data may be fatal in showing that the trend is persistent and not merely fortuitous, as required under CJEU case law.^186^ Moreover, the lack of a causal explanation for *why* women might be overrepresented in unpaid internships could further hinder the Principle 8 QFT claim. Whilst there is doubt over whether an explanation is required under the statistical approach,^187^ it could assist in bolstering the limited statistical evidence (in analogy with cases like *Bilka*, which referenced the ‘difficulties encountered by women workers in working full-time’^188^). Although tentative reasons, such as women being less likely to negotiate for payment^189^ or the prevalence of unpaid internships in gendered sectors, could be put forward,^190^ the absence of clear causation throws further doubt on the claim’s success.

An alternative approach could be to rely upon the CJEU’s expanding case law adopting a ‘common-sense’ view of establishing a particular disadvantage. In cases such as *Elsner-Lakeberg*,^191^  *Lufthansa*^192^ and *Rinke*,^193^ the CJEU argue that, where a rule, provision or policy has failed to consider the ‘greater burden’,^194^ ‘greater risk’^195^ or ‘greater difficulty’^196^ it puts on one group compared to another, it may give rise to indirect discrimination. In *Odar*, for example, the CJEU held that a compensation policy resulted in indirect disability discrimination for ‘disregarding the risks faced by severely disabled people, who generally face greater difficulties in finding new employment’.^197^ The CJEU’s growing discussion of ‘stigmatisation’ within the nexus of race and ethnic origin discrimination, seen in *CHEZ*^198^ and recently in *Slagelse*,^199^ can also be regarded as part of the common-sense approach, since the CJEU is examining the instinctive impact of a policy. The approach is perhaps most stark in cases concerning discrimination based on nationality under article 18 TEU, where, albeit in a different context, the CJEU adopts a ‘logical implication and risk assessment’ approach over exact statistical thresholds.^200^

The uniting feature of these cases is that the CJEU makes intuitive deductions about the disadvantageous effect of a policy rather than relying on statistics. Whilst this reasoning may build upon stereotypes,^201^ in some ways the common-sense approach is consequential development to the CJEU’s strict statistical approach: as societal awareness of structural discrimination expands, case law can make more ‘jumps’ in finding indirect discrimination without having to methodically evidence them.

Until recently, it was unclear when the CJEU would adopt the common-sense approach over the statistical approach—this was particularly true following *Voß*, in which the CJEU seemed to acknowledge both but endorse neither.^202^ However, following *IK v KfH*, the CJEU addressed the distinction more openly, stating that ‘statistical evidence is only one factor among others’ for establishing indirect sex discrimination and that, where other qualitative factors existed, there was ‘no need for there to be … considerably more men than women’ in the advantaged group.^203^ Whilst academic interpretation of the case has varied significantly,^204^ it is clear that the CJEU endorses a wider, common-sense understanding of what might constitute a particular disadvantage. For the purposes of a claim against Principle 8 QFT, this endorsement is critical.

It could be argued that Principle 8 QFT, in permitting unpaid internships, puts women at a particular disadvantaged compared to unpaid male interns due to the ‘greater risk’ they face of sexual harassment.^205^ The comparison here is between unpaid female and unpaid male interns, rather than a claim based on the bifurcation of paid and unpaid internships discussed earlier.

By adopting a common-sense approach, the CJEU is better able to consider the vulnerability that young female interns face, due to the factors inherent to an unpaid internship discussed earlier, than under the statistical approach. As Advocate General Ramos argued in *IK v KfH*, the approach ‘involves examining the labour market as a whole’,^206^ which would enable Principle 8 QFT to be considered against the backdrop of Europe’s gender pay gap and the already reported high rates of sexual harassment of interns. He also stated that it is ‘worth remembering the logic’ behind indirect discrimination, which is ‘characterised by the sole fact that persons of one sex are put at a particular disadvantage’ compared to the other.^207^ This reflects Advocate General Kokott’s Opinion in *CHEZ*, which argued that a measure does not have to ‘seriously’ disadvantage one group over another; rather, it must simply affect certain persons ‘more adversely’.^208^ Building on this, it can be argued that unpaid internships are liable to have a more adverse effect on women than men. Since women are more at risk of sexual harassment in the workplace, and the status of unpaid internships significantly exacerbates this risk of abuse for the reasons identified earlier, then a common-sense argument would conclude that they place a ‘greater burden’ or ‘risk’ on female interns relative to male interns.

If the common-sense approach is adopted, then the upshot of this ‘light touch’ analysis is that it puts more weight on the justification stage.^209^ Having examined the various justifications for Principle 8 QFT previously, this section will not repeat them here. It is worth noting though that, whilst student internships are excluded from the QFT, the fact that the additional pressure to complete an educational course combined with insufficient institutional oversight (since it is unclear who is responsible for the intern: the university or the employer) has been identified as an aggravating risk factor for sexual harassment^210^ is likely to be a consideration for future legislators.

More broadly, however, this test case demonstrates the importance of the EU’s gender mainstreaming obligations in anticipating these discriminatory implications *before* sufficient statistical evidence demonstrates the extent of the disadvantage. This speaks to wider, well-documented limitations of non-discrimination law, namely that it is aimed at providing remedies to existing discrimination (eg by waiting until sufficient studies show the scale of sexual harassment) rather than proactively preventing them, and that it is aimed predominantly at ensuring symmetry with male interns, rather than accommodating the vulnerabilities specific to women (eg that the status of an unpaid intern brings distinct risks of abuse).^211^

Adopted by the EU in the 1990s, gender mainstreaming was an attempt to address some of these concerns by ensuring that a gender perspective was applied to new policy initiatives.^212^ Known as the ‘mainstreaming clauses’,^213^ articles 8–10 TFEU require, *inter alia*, the EU to take gender equality into account when ‘defining and implementing its policies’. This commitment was reaffirmed in the EU’s most recent Gender Equality Strategy, which states that the Commission will ‘systematically includ[e] a gender perspective in all stages of policy design in all EU policy areas’.^214^

However, the Commission’s initial proposal for the QFT contained no mention of the impact of unpaid internships on women, nor do the Commission’s latest (2024) proposals.^215^ Despite being heralded as the EU’s ‘most promising approach’ to equal opportunities,^216^ the rigour with which it applied gender mainstreaming to its internship policy appears questionable. This is perhaps not surprising: whilst there is general academic agreement that the EU’s gender mainstreaming efforts started well,^217^ many argue this early enthusiasm has since lagged for a variety of reasons, notably budget constraints and the ill-defined concept of ‘gender equality’.^218^

One area which is, however, recognised for the impact of gender mainstreaming has been the EU’s initiatives on work–life balance, due to the awareness that non-discriminatory access to work is ‘an area which underpins and affects any other policy’.^219^ Given that internships equally concern *access* to the labour market, it can be argued that this is an area where similar attention under articles 8–10 TFEU ought to have been paid.

The extent to which the mainstreaming clauses are justiciable is unclear,^220^ although Psychogiopoulou’s review of the CJEU’s (limited) case law suggests that the provisions have been construed as imposing a legal duty on the EU legislator and that they have been successfully relied upon to supplement the central claim.^221^ It could therefore be argued that articles 8–10 TFEU were disregarded in the formation of the QFT as a potentially supplementary point to the claim that Principle 8 QFT results in indirect sex discrimination. The argument would be that the likelihood of unpaid internships exacerbating the existing gender discrepancies in pay, caregiving duties and sexual harassment ought to have been considered when the policy was drafted. This issue is no doubt likely to be of heightened significance if the new proposals on internships also fail to consider a gender perspective.

To conclude, female interns appear less likely to be paid and more likely to be sexually harassed than male interns. Whether these statistics are sufficiently serious enough to amount to sex discrimination is one issue; whether policy makers are obliged to consider these issues before making recommendations is another. As has been argued in this section, the chances of the former happening may have been lower had the latter occurred.

## 6. Conclusion

This article has sought to address persistent speculation that unpaid internships breach EU equality law on multiple grounds. The issue is not just that internships are unpaid, but that bifurcation between paid and unpaid creates a two-tiered entry system into the labour market which discriminates along protected grounds.

The arc of the article suggests that Principle 8 QFT could plausibly be found to discriminate based on social origin, age and sex under article 21 Charter. Such a finding, however, would not be without significant judicial hurdles. Whether EU recommendations ought to be reviewable; whether the so-far dormant ground of social origin discrimination should be awakened; whether the education-to-work transition is comparable to the work-to-retirement transition; and whether a ‘common-sense’ approach could be used to evidence indirect discrimination are some of the issues identified.

Against the backdrop of soaring internship rates and a general shift away from employer-funded training to worker-funded training,^222^ a ruling such as the one discussed in this article would have major ramifications on the existence of unpaid internships across Europe. Whilst focus was placed predominantly on unpaid open-market internships, a finding would inevitably question the legitimacy of unpaid student and professional internships, particularly given their porous boundaries with open-market internships. It would likely involve a re-evaluation of where internships sit within the framework of apprenticeships, volunteering and entry-level jobs. If there is a standout takeaway from the analysis, it is that the most fatal aspect of Principle 8 QFT appears to be its blanket allowance of unpaid internships, without targeted use. This suggests that tailored use may fare better under the proportionality test.

Reflecting more broadly, the analysis ultimately cast light on various limitations of equality law. The artificial separation of the grounds examined—social origin, age and sex—failed to capture the intersectional reality of a young working-class female intern experiencing discrimination on all fronts. The likely shift of debate from the discriminatory impacts of unpaid to *underpaid* internships, even if a ruling was made out, underscores the reactionary nature of discrimination law. Other grounds, such as race and ethnic origin, disability or discrimination based on employment status,^223^ could have been discussed, but this speaks to a wider call for holistic, proactive evaluation of policy. Most significantly, though, the difficulties and obstacles involved in bringing a claim testified to the need for clear, robust legislation. The CJEU is otherwise placed in the precarious position of deciding whether it is constitutional for the ‘bridge’ between education and work to be closed for a few if it lets the rest through.

## Footnotes

1 As outlined in-text below.

2 Commission, ‘Integration of Young People into the Labour Market’ (Eurobarometer 523, 2023) found 78% of the 18- to 35-year-olds interviewed completed at least one of the four types of internships, and the Commission expects internships to grow by 16% by 2030: Commission, ‘Commission Takes Action to Improve Traineeships’ (Press Release, 20 March 2024) <https://ec.europa.eu/commission/presscorner/detail/en/ip_24_1489≥ accessed 7 April 2025.

3 Council Recommendation on a Quality Framework for Traineeships [2014] OJ C88/01, recital 27.

4 The EU uses this categorisation, referring to internships as ‘traineeships’: Kari Hadjivassiliou and others, ‘Study on a Comprehensive Overview on Traineeship Arrangements in Member States’ (Commission 2012) 3; Commission, ‘Proposal for a Directive on Traineeships’ COM (2024) 132 final, 2 and 15. ILO publications also adopt this categorisation: see Andrew Stewart and others, *Internships, Employability and the Search for Decent Work Experience* (ILO and Edward Elgar 2021). For discussion on the difference with apprenticeships, see Joanna Helme, ‘The Problems and Paradoxes with the EU’s Regulation of Traineeships’ (2024) 53 ILJ 679.

5 Discussed further at n 35.

6 Eurobarometer (n 2): 55% are paid *or compensated*, meaning that ‘paid’ internships could be below national minimum wage.

7 Erica Holt-White and Carl Cullinane, ‘Unpaid and Underpaid Internships’ (Sutton Trust 2025) 10, focusing on open-market internships (although types are not specified).

8 Carl Cullinane and Rebecca Montacute, ‘Pay As You Go?’ (Sutton Trust 2018); Holt-White and Cullinane (n 7).

9 ‘Exploiting the Dream’ (National Union of Journalists 2019).

10 Paula McDonald, ‘Open Market Internships: What Do Intermediaries Offer?’ [2020] Journal of Education and Work 33. For an example, students at LSE can apply for the price of £650 to do an eight-week unpaid internship through the internship broker, Intern Group <www.lse.ac.uk/study-at-lse/study-abroad/the-general-course/internships> accessed 7 February 2026.

11 McDonald and others, ‘Challenging the Assumptions Supporting Work Experience as a Pathway to Employment’ in Stewart and others (n 4) 78; see also QFT (n 3) recital 2.

12 Eurostat, ‘Unemployment Statistics’ <https://ec.europa.eu/eurostat/statistics-explained/index.php?title=Unemployment_statistics> accessed 27 January 2026.

13 Niall O’Higgins and Luis Pinedo, ‘Interns and Outcomes: Just How Effective Are Internships as a Bridge to Stable Employment?’ (ILO 2018).

14 Emily O’Reilly, ‘Unpaid Internships Amount to Social Discrimination’ *The Parliament* (9 November 2018) <www.theparliamentmagazine.eu/news/article/unpaid-internships-amount-to-social-discrimination> accessed 7 April 2025.

15 ‘Good Work: The Taylor Review of Modern Working Practices’ (UK Government 2018) 91.

16 The United States’ main internships dataset, conducted annually with around 20,000 participants by the National Association of Colleges and Employers (NACE), consistently finds that Black and Hispanic students are ‘significantly underrepresented as a proportion of paid interns’ and less likely to do an internship than their white counterparts: ‘Unpaid Internships and the Need for Federal Action’ (NACE 2023) <www.naceweb.org/about-us/advocacy/position-statements/position-statement-us-internships> accessed 26 January 2026.

17 Julia López López, ‘Traineeships and Systemic Discrimination against Young Workers’ in Stewart and others (n 4) 322.

18 Eurobarometer (n 2); NACE (n 16).

19 Alysia Blackham, ‘Working at the Edges of Legal Protection’ in Stewart and others (n 4).

20 ibid.

21 There are two main supranational decisions on internships, neither of which directly addresses non-discrimination law: Case 454/2014/PMC *European External Action Service’s practice of offering unpaid traineeships in EU Delegations* [2017] Decision of European Ombudsman Emily O’Reilly; *European Youth Forum (YFJ) v Belgium* Complaint No 150/2017, European Committee of Social Rights Decision on Merits, 2022.

22 The UK Parliament website states that MPs ‘may take on volunteers and unpaid interns’ <www.parliament.uk/about/careers/work-mp/> accessed 7 April 2025.

23 Sarah Butler, ‘Initiative to Crack Down on Unpaid Internships Launched in UK’ *The Guardian* (9 February 2018) <www.theguardian.com/society/2018/feb/08/initiative-to-crack-down-on-unpaid-internships-launched-in-uk> accessed 2 April 2025.

24 European Parliament Resolution with Recommendations on Quality Traineeships in the Union [2023] OJ C/2024/484, para C.

25 QFT (n 3).

26 In English. Helme (n 4); Annika Rosin, ‘Labour Law Protection of Trainees’ (PhD Thesis, University of Turku 2017), which is comprised of four articles, predominantly examining the regulation of interns in Estonia, France and Finland. See also Kornélia Kerti, ‘The Right to Fair Remuneration and Unpaid Internships in the European Union: Decent Work for Interns’ (Masters Thesis, Tilburg University 2020); for a brief discussion of the status of interns, see Martin Risak and Thomas Dullinger, *The Concept of ‘Worker’ in EU Law* (ETUI 2018).

27 Stewart and others (n 4); Andrew Stewart and Rosemary Owens, ‘Work at the Intersection of Employment, Education, Training, and Volunteering’ in Guy Davidov and others (eds), *Oxford Handbook of the Law of Work* (OUP 2024); Rosemary Owens and Andrew Stewart, ‘Regulating for Decent Work Experience’ (2016) ILR 679.

28 The main UK publications are non-legal reports by non-profit organisations: Sutton Trust (nn 7 and 8); Institute for Public Policy Research, ‘The Inbetweeners: The New Role of Internships in the Graduate Labour Market’ (2017).

29 The 2023 and 2013 Eurobarometer surveys (n 2) are the main EU source; most UK data is non-governmental (n 28) and there appears to be no ILO data collection.

30 QFT (n 3).

31 Charter of Fundamental Rights of the European Union [2016] OJ C202/02.

32 C-236/09 *Test-Achats v Conseil des Ministers* ECLI:EU:C:2011:100, which held that a provision in an EU directive was invalid for discriminating contrary to arts 21 and 23 Charter. Whilst the CJEU refers to the Treaties as ‘constitutional’, it uses the term ‘invalid’ rather than unconstitutional: C-294/83 *Les Verts v European Parliament* ECLI:EU:C:1986:166, para 23.

33 QFT, recital 28.

34 ALMP internships are best associated with ‘workfare’ schemes, where the individual receives unemployment benefits during the work placement, which may be topped up by the internship provider: see Hadjivassiliou and others (n 4); Amir Paz-Fuchs, ‘Trainees—the New Army of Cheap Labour: Lessons from Workfare’ in Andrew Stewart and others (n 4); Irene Nikoloudakis, ‘Regulating Internships in Active Labour Market Programmes’ in Stewart and others (n 4); Paz-Fuchs and Eleveld, ‘Workfare Revisited’ (2016) 45 ILJ 29.

35 Examples include the university offering summer internships, which are not strictly ‘part of’ a course, or where students are required to complete an internship but must source it from the open market, as reflected in C-344/20 *LF v SCRL* ECLI:EU:C:2022:77. See Helme, ‘The Problems and Paradoxes’ (n 4); Joanna Helme, ‘Regulating Traineeships in the EU’ (European Parliament, 2025). For discussion on how universities have increasingly ‘promoted or endorsed’ open-market internships in fields with ‘no statutory or professional requirements’, see McDonald (n 10) 33; Deanna Grant-Smith and Paula McDonald, ‘Ubiquitous yet Ambiguous: An Integrative Review of Unpaid Work’ (2018) 20 IJMR 559.

36 Commission, ‘Proposal for a Recommendation on a Reinforced Quality Framework for Traineeships’ COM (2024) 133 final; Commission, COM (2024) 132 final (n 4).

37 See n 26.

38 Predominantly due to the EU competency restrictions around pay and education, see Helme, ‘Regulating Traineeships’ (n 35).

39 QFT (n 3).

40 Commission, COM (2024) 132 final (n 4); Commission, COM (2024) 133 final (n 36).

41 As discussed (n 34), legitimising unpaid ALMP internships was not itself as controversial.

42 Consolidated Version of the Treaty on the Functioning of the European Union [2012] OJ C326/47.

43 C-16/16 *Belgium v Commission* ECLI:EU:C:2018:79, para 29.

44 Whilst case law is limited, these appear to be the main criteria: Anthony Arnull, ‘EU Recommendations and Judicial Review: Belgium v Commission’ (2018) 14 European Competition Law Review 609.

45 Commission, ‘Consultation Document under Article 154 TFEU on Traineeships’ COM (2023) 4785 final, 19.

46 C-322/88 *Grimaldi v Fonds* EU:C:1989:646, para 18.

47 Remarks in *Rinke* could support this, which state that compliance with the prohibition on sex discrimination is a ‘condition governing the legality of *all* measures adopted by the Community’: C-25/02 *Rinke v Ärztekammer* ECLI:EU:C:2003:435, paras 25–8.

48 arts 165(4) and 166(4) TFEU do not allow for directives on education and vocational training, respectively.

49 C-300/89 *Commission v Council* ECLI:EU:C:1991:115, Opinion of AG Tesauro, I-2879. See also C-626/15 and C-659/16 *Commission v Council* ECLI:EU:C:2018:362, Opinion of AG Kokott, para 81.

50 Helme, ‘Regulating Traineeships’ (n 35).

51 C-188/91 *Shell v Hauptzollamt* ECLI:EU:C:1993:24, para 18.

52 C-16–65 *Schwarze v Einfuhr-und-Vorratsstelle* ECLI:EU:C:1965:117, although art 267 cannot be used solely to circumvent the time limits in art 263: C-188/92 *TWD v Bundesrepublik* ECLI:EU:C:1994:90.

53 C-293/12 and C-594/12 *Digital Rights Ireland v Minister for Communications* ECLI:EU:C:2014:238.

54 C-92/09 and C-93/09 *Volker v Hessen* ECLI:EU:C:2010:662.

55 C-362/14 *Schrems v Data Protection Commissioner* ECLI:EU:C:2015:650.

56 The terms are nonetheless distinct, discussed in-text below. For an outline, see Angelo Capuano, *Class and Social Background Discrimination in the Modern Workplace* (BUP 2023) ch 2; Council of Europe, ‘Tackling Discrimination Based on Social Origin’ (Report 15499, 2022).

57 As stated in Shreya Atrey, ‘The Intersectional Case of Poverty in Discrimination Law’ (2018) Human Rights Law Review 411, 411; Kate Malleson, ‘Equality Law and the Protected Characteristics’ (2018) 4 MLR 598, 611; Alex Benn, ‘The Big Gap in Discrimination Law: Class and the Equality Act 2010’ (2020) 3(1) University of Oxford Human Rights Hub Journal 30, 30. Reasons given include difficulties in defining social origin, reliance on positive duties and risks of rending positive discrimination efforts unlawful: Lizzie Barmes and Kate Malleson, ‘The Case for Caution in Making Social Class a Protected Characteristic’ (Oxford Human Rights Hub 2023) <https://ohrh.law.ox.ac.uk/the-case-for-caution-in-making-social-class-a-protected-characteristic-under-the-uks-equality-act-2010/> accessed 3 January 2026.

58 Despite most supranational human rights instruments recognising social origin as a ground, including the Universal Declaration of Human Rights, art 2; European Convention of Human Rights, art 14; ILO Convention 111 on Discrimination (Employment and Occupation), art 1. For an analysis of the very limited case law, see Council of Europe (n 56); Capuano (n 56); Geraldine Van Bueren, ‘Inclusivity and the law: Do we need to prohibit class discrimination?’ (2021) 3 EHRLR 274.

59 Only Croatia, Czech Republic, Hungary, Romania and Slovakia do, see Council of Europe (n 56).

60 QFT (n 3) recital 5.

61 European Parliament Resolution (n 24) para 6.

62 Barmes and Malleson (n 57); Van Bueren (n 58).

63 There appear to be no CJEU decisions on social origin, see EU Agency for Fundamental Rights (FRA), *Handbook on European Non-Discrimination Law* (EU 2018) 216; Anne-Marie Cotter, *Class Act: An International Perspective on Class Discrimination* (Routledge, 2011); the Explanations relating to the Charter of Fundamental Rights of the European Union [2007] OJ C303 do not provide further clarity.

64 Since art 52(3) Charter requires CJEU interpretation to be in line with the ECHR, and the Charter Explanations (n 63) state that art 21 applies in compliance with art 14 ECHR.

65 Parliamentary Assembly Council of Europe Resolution 2432 ‘Tackling Discrimination Based on Social Origin’ (adopted 26 April 2022), para 1.

66 ibid para 2.

67 Birth, social origin and property are interconnected according to FRA (n 63) and Committee on Economic, Social and Cultural Rights (CESCR), ‘General Comment No 20: Non-discrimination’ (Doc E/C.12/GC/20, 2009) para 24.

68 Noting that property is a ‘broad’ ground which includes ‘a lack of’ personal property including income: CESCR (n 67) para 25.

69 As alluded to in Barmes and Malleson (n 57).

70 Consolidated Version of the Treaty on European Union [2002] OJ C325/5 (TEU), art 3; TFEU, art 9.

71 Van Bueren (n 58) 280; this ties into Collins’s work on realigning equality law as focused on social inclusion: Hugh Collins, ‘Discrimination, Equality and Social Inclusion’ (2003) 66 MLR 16.

72 C-336/19 *Centraal Israëlitisch and Others* ECLI:EU:C:2020:1031, para 77.

73 Directive 2000/78/EC establishing a general framework for equal treatment in employment and occupation [2000] OJL303, art 2.

74 As discussed in Jeremias Adams-Prassl and others, ‘Directly Discriminatory Algorithms’ (2023) 86 MLR 144; Hugh Collins and Tarunabh Khaitan, *Foundations of Indirect Discrimination Law* (Hart Publishing 2020); Sandra Fredman, *Discrimination Law* (OUP 2022) ch 4.

75 The Sutton Trust’s study of 614 participants found those with working class backgrounds were 20 percentage points less likely to take part in unpaid internships than middle class interns, Sutton Trust (n 7) 8; a study of 3800 respondents found a ‘clear access gap’ based on lower socio-economic status, Damian Oliver and others, ‘Unpaid Work Experience in Australia’ (Department of Employment, 2016) 60; a review of studies found that social class negatively affected access to internships, McDonald and others (n 11); and the Fair Internship Initiative (FII) found 83.5% of unpaid interns would have been unable to afford the internship without their family’s economic support, FII, ‘UN Internships Report’ (2017) 5.

76 Sutton Trust (n 7). Another survey found that 91% of university students believed connections were most important in securing internships: Blackham (n 19).

77 While other factors arising from social origin, like parental education, also affect the likelihood of completing internships (see McDonald and others (n 11)), the financial barrier has been identified as most prohibitive: Sutton Trust (n 7) 15.

78 European Ombudsman (n 21) para 5.

79 C-16/19 *VL v Szpital Kliniczny* ECLI:EU:C:2021:64, paras 44 and 46; C-335/11 and C-337/11 *Ring v Dansk; Werge v Pro Display* ECLI:EU:C:2013:222, para 74; C-499/08 *Ingeniørforeningen I Danmark*EU:C:2010:600 paras 23–4; C-406/15 *Milkova v Izpalnitelen* EU:C:2017:198 para 42.

80 C-83/14 *CHEZ v Komisia* ECLI:EU:C:2015:480, para 76.

81 C-417/23 *Slagelse Almennyttige v MV* ECLI:EU:C:2025:1017, para 92.

82 See Adams-Prassl and others (n 74).

83 Council of Europe Resolution (n 65) part B, para 1.

84 As evidenced in n 75.

85 See n 74.

86 art 52 states that limitations on Charter freedoms must be ‘necessary and genuinely meet objectives of general interest’: the CJEU in *Test-Achats* relied on this although direct discrimination is not normally justifiable, likely because there is no secondary legislation to defer to: Denis Martin, ‘Article 21’ in Manuel Kellerbauer and others (eds), *The EU Treaties and the Charter of Fundamental Rights* (OUP 2019).

87 Directive 2000/78/EC (n 73) art 6.

88 QFT (n 3) recitals 1–2 and Principles 1 and 3; see also *YFJ v Belgium* (n 21) para 100; Commission, COM (2024) 132 final (n 4) 3.

89 C-143/16 *Abercrombie v Bordonaro* ECLI:EU:C:2017:566; C-88/08 *Hütter v Universität* ECLI:EU:C:2009:381.

90 Rachel Horton, ‘Justifying Age Discrimination in the EU’ in Uladzislau Belavusau and Kristin Henrard (eds), *EU Anti-Discrimination Law beyond Gender* (Hart Publishing 2018) 280.

91 Nearly half of British employers believe graduates without job experience have ‘no or little chance’ of being offered a graduate job: McDonald and others (n 11) 78.

92 ibid; Niall O’Higgins and Luis Pinedo Caro, ‘What Makes for a “Good” Internship?’ in Stewart and others (n 4); O’Higgins and Pinedo’s review of studies showed paid internships had ‘overwhelmingly’ better outcomes than unpaid, O’Higgins and Luis Pinedo, ‘Interns and Outcomes’ (n 13) 25; the Eurobarometer (n 2) found 74% of paid interns were subsequently employed by the traineeship provider compared to 53% of unpaid interns; 72% of paid trainees agreed that their traineeship was helpful for finding a regular job compared to 57% unpaid trainees; NACE (n 16) also found paid interns received more job offers than unpaid interns and a higher starting salary.

93 Paz-Fuchs (n 34) 11; Nikoloudakis (n 34) 253.

94 Paz-Fuchs (n 34).

95 McDonald and others’ review of studies found ‘only tenuous and incomplete support’ for work experience delivering real employment benefits, and noted that as internships become more ubiquitous, the less value added they give individual candidates, McDonald and others (n 11) 84; Holford found unpaid internships led to worse employment outcomes three and half years later compared to those who went straight into paid work, Angus Holford, ‘Access to and Returns from Unpaid Graduate Internships’ (IZA Paper 2017); see also n 92.

96 QFT (n 3) recital 4.

97 Eurobarometer (n 2).

98 C-388/07 *Age Concern v Secretary of State* ECLI:EU:C:2009:128, para 51; C-167/97 *Regina v ex parte Seymour-Smith and Perez* ECLI:EU:C:1999:60, para 76; C-159/10 and C-160/10 *Fuchs and Köhler v Hessen* ECLI:EU:C:2011:508, paras 76–83.

99 Paz-Fuchs (n 34).

100 See eg claims in the UK that minimum wage legislation would destroy up to 2,000,000 jobs, which did not transpire: Institute for Government, ‘The Introduction of the National Minimum Wage 1998’ (2012) 62.

101 C-341/08 *Petersen v Berufungsausschuss* ECLI:EU:C:2010:4.

102 As promoted by the Sutton Trust (n 7).

103 ibid.

104 McDonald and others (n 11).

105 There is significant scholarship on the distinctiveness of employment contracts compared to other contracts in determining an individual’s life fulfilment, see Virginia Mantouvalou (ed), *The Right to Work* (Bloomsbury 2015); the importance of the right to work for participation in ‘economic, social and cultural life’ was also acknowledged in *Fuchs* (n 98) para 64.

106 Council of Europe (n 56) para 52: access to ‘higher’ professions is a key indicator of social mobility.

107 Fabrizio Bernardi and Gabriele Ballarino (eds), *Education, Occupation and Social Origin* (Edward Elgar 2016) 1.

108 For a summary, see Alexi Gugushvili and others, ‘The Direct Effect of Social Origins on Social Mobility Chances’ (2017) 33 ESR 305.

109 For example, art 14 Charter enshrines the right to free compulsory education, and the Explanations (n 63) states that the EU’s training policies must respect free compulsory education.

110 Eurobarometer (n 2) 23.

111 These are one of the most popular in the UK: Sutton Trust (n 7).

112 Council of Europe Resolution (n 65).

113 European Ombudsman (n 21) para 11.

114 C-432/14 *O v Bio Philippe* ECLI:EU:C:2015:643.

115
*Abercrombie* (n 89).

116 C-46/17 *Hubertus v Bremen* ECLI:EU:C:2018:131.

117 Opinion of AG Kokott in *Test-Achats* (n 32) para 50.

118 Justifications for this ‘special’ treatment of age tend to be grouped into the ‘complete life view’ and the ‘fair innings’ argument, although it is not clear which the CJEU adopts: see Horton (n 90), who challenges both justifications. Arguing against the specialist treatment, see Elaine Dewhurst, ‘Is Age Exceptional? Challenging Existing Rationales and Exploring Realities’ (2023) 36 Harv Hum Rts J 247; Pnina Alon-Schenker, ‘Age is Different: Revisiting the Contemporary Understanding of Age Discrimination’ (2013) 17 Canadian Labour and Employment Law Journal 31; Beryl Haar, ‘EU Age Discrimination Law: A Curse or a Blessing for EU Youth Policy?’ in Belavusau and Henrard (n 90).

119 Directive 2000/78/EC (n 73) art 6.

120 Recital 4 of the draft Traineeship Directive (n 4) states traineeships are useful ‘for persons of any age’, and traineeships with the Commission and Council are open to ‘all EU citizens, regardless of age’: see <https://traineeships.ec.europa.eu/who-can-apply> and <www.consilium.europa.eu/en/general-secretariat/jobs/traineeships/> accessed 7 April 2025.

121 Commission, COM (2023) 4785 final (n 45) 9.

122 David Yamada, ‘The Employment Law Rights of Student Interns’ (2002) 35 CLR 215.

123 Eurobarometer (n 2) 1.

124 Regulation 508/2014 on the European Maritime and Fisheries Fund [2014] OJ L149/1, art 29 (3).

125 Many have commented on the CJEU’s ‘striking’ use of proxies in age discrimination, notably as a proxy for seniority, employment prospects and previous experience, see Horton (n 90) 283; Marie Mercat-Bruns, ‘The Ambivalent Treatment of Age Discrimination’ in Colm O’Cinneide and others, *Research Handbook on European Anti-Discrimination Law* (Edward Elgar 2025); Colm O’Cinneide, ‘The Growing Importance of Age Equality’ (2013) 11 The Equal Rights Review 99; Colm O’Cinneide, ‘Age Discrimination and the European Court of Justice: EU Equality Law Comes of Age’ (2010) 2 Revue des Affaires Européennes 253.

126 C-555/07 *Kücükdeveci v Swedex* ECLI:EU:C:2010:21, para 40.

127
*Hütter* (n 89) para 48.

128 Directive 2000/78/EC (n 73) art 2.

129 See nn 92 and 95.

130 Two claims were brought before the Massachusetts Commission Against Discrimination against a magazine for denying mature students internships because of their age: Yamada (n 122).

131
*Hubertus* (n 116).

132 C-250/09 and C-268/09 *Georgiev v Universitet* ECLI:EU:C:2010:699.

133
*Abercrombie* (n 89) Opinion of AG Bobek.

134 ibid para 53.

135 ibid para 56.

136 ibid paras 70–1.

137 As outlined in Beryl Haar’s analysis of the CJEU age discrimination case law, notably in C-144/04 *Mangold v Helm* ECLI:EU:C:2005:709, para 64, which regarded the ‘benefit of stable employment’ as a ‘major element in the protection of workers’: see Beryl Haar, ‘Is the CJEU Discriminating in Age Discrimination Cases?’ (2020) 13 ELR 78.

138 Primarily because it reinforces the formal, ‘like for like’ concept of equality by comparison rather than addressing unique challenges: see Nozizwe Dube, ‘A Typology of Comparators and Comparisons in EU Equality Law’ [2025] CMLR 49.

139
*O* (n 114).

140 C-313/02 *Wippel v Peek* ECLI:EU:C:2004:607.

141 As discussed by AG Bobek in *Abercrombie* (n 89).

142
*Mangold* (n 137) para 59.

143
*Hubertus* (n 116) para 29.

144 QFT (n 3) recital 27.

145 C-548/15 *de Lange v Staatssecretaris* ECLI:EU:C:2016:850.

146
*O* (n 114) paras 36 and 38.

147
*Abercrombie* (n 89) paras 33, 42 and 44.

148 Directive 2000/78/EC (n 73), art 6(1)(a).

149 The CJEU simply stated that the contested measure in *Hütter* (the exclusion of work prior to 18 years old from length of service calculations) ‘can be distinguished’ from lower wages for young people: *Hütter* (n 89) para 49.

150
*Abercrombie* (n 89) para 31; *Kücükdeveci* (n 126) para 38; *Mangold* (n 137) para 63.

151 Mark Freedland and Lucy Vickers, ‘Age Discrimination and EU Labour Rights Law’ in Alan Bogg and others (eds), *Research Handbook on EU Labour Law* (Edward Elgar 2016) 534 and 544, notably in cases relating to national retirement ages and redundancy criteria.

152 Horton (n 90) 533.

153 O’Cinneide, ‘Age Discrimination’ (n 125) 6.

154 Horton (n 90) 290.

155 See n 98; see also Horton (n 90) 281, who highlights the CJEU’s willingness in age discrimination cases to ‘reject measures based on poorly evidenced or inconsistent claims’.

156 Seen in *Kücükdeveci* (n 126) para 42 (comparing young people who enter active life early with those who take longer periods of training); *Hütter* (n 89) para 46 (comparing unequal treatment between young people in general education compared to those in vocational education).

157 Globally, women perform three times more unpaid care work than men: Commission, ‘Peer Review on “Work–Life Balance: Promoting Gender Equality”’ (EU 2022).

158 Eurobarometer (n 2).

159 The UK’s Sutton Trust found that among female interns, 72% completed an unpaid or underpaid internships compared to 66% of male interns, Sutton Trust (n 7) 25; Oliver and others (n 75) found men in Australia (26.4% points) were more likely than women (16.3% points) to receive financial compensation; Blackham (n 19).

160 Karly Wildenhaus, ‘Wages for Intern Work: Denormalizing Unpaid Positions in Archives and Libraries’ (2019) 2 Journal of Critical Library and Information Studies 1, 6.

161 Blackham (n 19) 305; Helme, ‘The Problems and Paradoxes’ (n 4).

162 Since remuneration is one of the criteria for worker status: C-66/85 *Lawrie-Blum v Baden-Württemberg* ECLI:EU:C:1986:284, para 17.

163 See nn 92 and 95.

164 Helme, ‘The Problems and Paradoxes’ (n 4).

165 ILO, ‘Experiences of Violence and Harassment at Work’ (ILO 2022) 8.

166 As found in Dubourdieu-Rayrot and others’ study of nearly 300 accounts from student interns, combined with 63 court cases: Camille Dubourdieu-Rayrot and others, ‘Internships and Sexual Harassment: How the Status of Interns Reinforces Vulnerability’ (2025) 32 Organization 1061, 1061. See also Joseph La Lopa and Zilan Gong, ‘Sexual Harassment of Hospitality Interns’ (2020) 32 Journal of Hospitality & Tourism Education 88; Blackham (n 19); Elena Frank and others, ‘Trends in Sexual Harassment Prevalence and Recognition During Intern Year’ [2024] 5 JAMA; Anne Hewitt and others, ‘Are Work Experience Participants Protected against Sex Discrimination or Sexual Harassment?’ (2021) 46 ALJ 115.

167 Including the potential exclusion of interns from harassment and discrimination protection, discussed by Blackham (n 19); Hewitt and others (n 166).

168 Cynthia Bowman and Marybeth Lipp, ‘Legal Limbo of the Student Intern’ (2000) 23 Harv Women’s LJ 95; Hewitt and others (n 166); Kavita Kalsi, ‘Sexual Exploitation and Harassment in Labour Market Transitions’ (UKAid Department 2023); Blackham (n 19); Helme, ‘The Problems and Paradoxes’ (n 4).

169 Frank and others (n 166).

170
*Wang v Phonexic Satellite, Inc* (SDNY 2013).

171
*X v Mid Sussex Citizens Advice Bureau* [2012] UKSC 59, para 20.

172 The main examples include C-96/80 *Jenkins v Kingsgate* ECLI:EU:C:1981:80; C-70/84 *Bilka-Kaufhaus v Weber* ECLI:EU:C:1986:204; C-300/06 *Voß v Land Berlin* ECLI:EU:C:2007:757; C-385/11 *Moreno v INSS and TGSS* ECLI:EU:C:2012:746; C-243/95 *Hill and Stapleton v Revenue Commissioners* ECLI:EU:C:1998:298.

173 For an example which attempts to discern the ‘best approach’ to comparing statistics in light of complex case law, see *Voß* (n 172) para 41.

174 TFEU, art 157; see also Directive 97/81/EC concerning part-time work [1997] OJ L 14/9 and Directive 2006/54/EC on equal opportunities and equal treatment of men and women (recast) [2006] OJ L204/23, respectively.

175 Building upon a footnote which refers to the CJEU’s ‘common-sense’ approach regarding part-time workers in Mark Bell, ‘The Concept of Indirect Discrimination’ in O’Cinneide and others (n 125) 155.

176 ibid; Catherine Barnard, *EU Employment Law* (OUP 2012) ch 6.

177 The phrase ‘substantially higher proportion’ was changed to ‘particular disadvantage’ in Directive 2006/54/EC (n 173) and Directive 2000/78/EC (n 73): Sandra Fredman, ‘Reviving Indirect Discrimination: Essop v Home Office’ (2016) 8 The UK Supreme Court Yearbook 154.

178 C-184/22 and C-185/22 *IK v KfH* ECLI:EU:C:2024:637, Opinion of AG Ramos, para 37.

179
*Seymour-Smith* (n 98).

180
*Jenkins* (n 172).

181
*Bilka* (n 172).

182 C-226/98 *Jørgensen v Foreningen* ECLI:EU:C:2000:191.

183 C-405/20 *EB v BVAEB* ECLI:EU:C:2022:347.

184
*Seymour-Smith* (n 98) paras 60–1: the difference between these two statistical approaches is discussed in Barnard (n 176) 333.

185 As summarised by AG Léger’s Opinion, paras 57–8 in Case C-317/93 *Inge Nolte v Hannover* ECLI:EU:C:1995:154; see also FRA (n 63) para 6.3.

186 C-127/92 *Enderby v Frenchay* ECLI:EU:C:1993:859, para 17; *Seymour-Smith* (n 98) para 55.

187 Dagmar Schiek, ‘Indirect Discrimination’ in Dagmar Schiek and others (eds), *Cases, Materials and Text on Non-Discrimination Law* (Hart Publishing 2007) 385.

188
*Bilka* (n 172) para 29.

189 Arianne Barzilay, ‘Discrimination without Discriminating: Learned Gender Inequality’ (2019) 28 Cornell J L & Pub Pol’y 545.

190 Blackham (n 19).

191 C-285/02 *Elsner-Lakeberg v Nordrhein-Westfalen* ECLI:EU:C:2004:320.

192 C-660/20 *MK v Lufthansa* ECLI:EU:C:2023:789.

193
*Rinke* (n 47).

194
*Elsner-Lakeberg* (n 191) para 17, in relation to overtime for part-time work; *Lufthansa* (n 191) paras 48 and 63, referring to the ‘non-work-related burdens’ that part-time workers face.

195
*Ring and Werge* (n 79) para 76.

196 C-152/11 *Odar v Baxter* ECLI:EU:C:2012:772, para 70; *Ring and Werge* (n 79) para 91.

197
*Odar* (n 196) para 69.

198
*CHEZ* (n 80) para 87.

199
*Slagelse* (n 81) para 126.

200 Sara Hungler, ‘Indirect Discrimination and the Gendered Realities of Part-Time Work: IK v KfH’ (2025) 16 ELLJ 297, 305, relying particularly on the free movement of people cases of *O’Flynn* and *Masgio*.

201 Bell (n 175) 156–7; see also Schiek (n 187).

202 The judgment, concerning pay supplements for overtime, referred to both *Elsner-Lakeberg* and the earlier, largely contradictory decision of *Helmig*, but did not reconcile them: for discussion, see Marianne Hotvedt, ‘Substantive Equality in Part-Time Work: The Evolving Protection of the Directive’ (2025) 16 ELLJ 343.

203
*IK v KfH* (n 178) paras 63–7.

204 For example, Hotvedt (n 202) argues that the case endorses the greater burden test in *Elsner-Lakenberg* and thus upholds a substantive model of equality, while Hungler (n 200) argues the opposite; Qayyum meanwhile argues the decision clarified little: Faris Qayyum, ‘The Court of Justice’s Missed Opportunity for Clarity and Progress—IK v KfH’ (2025) 50 ELR 208.

205 There is scope to argue that unpaid internships also put a ‘greater burden’ on women due to their disproportionate caregiving duties, particularly interns in the later stages of the 18–35 age bracket; however, this concern features less prominently than sexual harassment in literature.

206
*IK v KfH* (n 178) AG Ramos para 47.

207 ibid paras 48–9.

208
*CHEZ* (n 80) para 93, discussed in Bell (n 175) 137.

209 Bell (n 175) 156.

210 Hewitt and others (n 166); Dubourdieu-Rayrot and others (n 166).

211 For seminal critiques, see Sandra Fredman, *Women and the Law* (Clarendon Press 1997) ch 9; Fredman, *Discrimination Law* (n 74).

212 Commission, ‘Incorporating Equal Opportunities for Women and Men into Community Policies and Activities’ COM (96) 67 final.

213 Evangelia Psychogiopoulou, ‘The Horizontal Clauses of Articles 8–13 TFEU’ (2022) 7 European Papers 1357, 1358.

214 Commission, ‘A Union of Equality: Gender Equality Strategy 2020–2025’ COM (2020) 152 final, reaffirmed for post-2025 in Commission, ‘A Roadmap for Women’s Rights’ COM (2025) 97 final, 4.

215 Commission, ‘Proposal for a Recommendation on a Quality Framework for Traineeships’ COM (2013) 857 final; Commission, COM (2024) 132 final (n 4); Commission, COM (2024) 133 final (n 36).

216 Mark Pollack and Emily Hafner-Burton, ‘Mainstreaming Gender in the EU’ (2000) 7 Journal of European Public Policy 432, 434.

217 Elaine Weiner, ‘A Missing Piece? Men and the Puzzle of Gender Mainstreaming’ (2023) 26 Men and Masculinities 604; Pollack and Hafner-Burton (n 216).

218 Vera Lomazzi and Isabella Crespi, *Gender Mainstreaming and Gender Equality in Europe *(Policy Press 2019) 146–7; Sophie Jacquot, ‘A Policy in Crisis’ in Emanuela Lombardo and Johanna Kantola (eds), *Gender and the Economic Crisis in Europe* (Springer 2017). Other reasons include lack of political will, reliance on soft obligations and the ‘integration’ of gender into policies without fundamentally rethinking the policy itself: Weiner (n 217); Pollack and Hafner-Burton (n 216); Teresa Rees, *Mainstreaming Equality in the EU* (Routledge 1998).

219 Eugenia Caracciolo di Torella, ‘The Principle of Gender Mainstreaming’ in Maria Bartoloni and others (eds), *The EU and the Proliferation of Integration Principles* (Routledge 2019) 47.

220 Caracciolo di Torella regards them as ‘relatively underexplored by legal commentators’: ibid 47.

221 Psychogiopoulou (n 213); key remarks include C-620/18 *Hungary v Parliament and Council* ECLI:EU:C:2020:1001, para 46; C-201/15 *AGET v Ypourgos* ECLI:EU:C:2016:972, para 78; *Test-Achats* (n 32) paras 19–20; C-414/16 *Egenberger v Evangelisches* ECLI:EU:C:2017:85, Opinion of AG Tanchev, para 93.

222 Stewart and others (n 4) 5.

223 As argued in *YFJ v Belgium* (n 21).

